# Identification of Genome-Wide Variants and Discovery of Variants Associated with *Brassica rapa* Clubroot Resistance Gene *Rcr1* through Bulked Segregant RNA Sequencing

**DOI:** 10.1371/journal.pone.0153218

**Published:** 2016-04-14

**Authors:** Fengqun Yu, Xingguo Zhang, Zhen Huang, Mingguang Chu, Tao Song, Kevin C. Falk, Abhinandan Deora, Qilin Chen, Yan Zhang, Linda McGregor, Bruce D. Gossen, Mary Ruth McDonald, Gary Peng

**Affiliations:** 1 Saskatoon Research Centre, Agriculture and Agri-Food Canada, Saskatoon, Saskatchewan, Canada; 2 Department of Plant Agriculture, University of Guelph, Guelph, Ontario, Canada; Chungnam National University, REPUBLIC OF KOREA

## Abstract

Clubroot, caused by *Plasmodiophora brassicae*, is an important disease on *Brassica* species worldwide. A clubroot resistance gene, *Rcr1*, with efficacy against pathotype 3 of *P*. *brassicae*, was previously mapped to chromosome A03 of *B*. *rapa* in pak choy cultivar “Flower Nabana”. In the current study, resistance to pathotypes 2, 5 and 6 was shown to be associated with *Rcr1* region on chromosome A03. Bulked segregant RNA sequencing was performed and short read sequences were assembled into 10 chromosomes of the *B*. *rapa* reference genome v1.5. For the resistant (R) bulks, a total of 351.8 million (M) sequences, 30,836.5 million bases (Mb) in length, produced 120-fold coverage of the reference genome. For the susceptible (S) bulks, 322.9 M sequences, 28,216.6 Mb in length, produced 109-fold coverage. In total, 776.2 K single nucleotide polymorphisms (SNPs) and 122.2 K insertion / deletion (InDels) in R bulks and 762.8 K SNPs and 118.7 K InDels in S bulks were identified; each chromosome had about 87% SNPs and 13% InDels, with 78% monomorphic and 22% polymorphic variants between the R and S bulks. Polymorphic variants on each chromosome were usually below 23%, but made up 34% of the variants on chromosome A03. There were 35 genes annotated in the *Rcr1* target region and variants were identified in 21 genes. The numbers of poly variants differed significantly among the genes. Four out of them encode Toll-Interleukin-1 receptor / nucleotide-binding site / leucine-rich-repeat proteins; *Bra019409* and *Bra019410* harbored the higher numbers of polymorphic variants, which indicates that they are more likely candidates of *Rcr1*. Fourteen SNP markers in the target region were genotyped using the Kompetitive Allele Specific PCR method and were confirmed to associate with *Rcr1*. Selected SNP markers were analyzed with 26 recombinants obtained from a segregating population consisting of 1587 plants, indicating that they were completely linked to *Rcr1*. Nine SNP markers were used for marker-assisted introgression of *Rcr1* into *B*. *napus* canola from *B*. *rapa*, with 100% accuracy in this study.

## Introduction

Clubroot disease, caused by *Plasmodiophora brassicae* Woronin, can result in a 10–15% reduction in seed yield globally on *Brassica* species and related crops [1]. The pathogen belongs to the Infra Kingdom Rhizaria, which is a diverse group of amoeboid protists [2]. The characteristic symptom of clubroot is the development of large, disorganized growths (clubs) on the roots of susceptible plants, leading to wilting, stunted growth, and premature ripening [3]. The life cycle of *P*. *brassicae* consists of a primary phase in root hairs and a secondary phase in the cortex and stele [4].

On the Canadian prairies, clubroot was first identified on canola (*B*. *napus* L.) in 2003 and has spread rapidly. Genetic resistance is considered to be the most effective means for clubroot management [5–7]. Identification and genetic mapping of clubroot resistance genes have been carried out in *B*. *rapa* [8–23], *B*. *oleracea* [24–32] and *B*. *napus* [33–36]. It has also been assessed in *Arabidopsis thaliana* [37] and *Raphanus sativus* [38]. Two resistant genes, *CRa* and *Crr1*, have been isolated from Chinese cabbage lines of *B*. *rapa*. They encode Toll-Interleukin-1 receptor / nucleotide-binding site / leucine-rich-repeat (TIR-NBS-LRR; TNL) proteins [39, 40].

Lines of *B*. *rapa* with resistance to several pathotypes of *P*. *brassicae* [41] have been identified [42, 43] and could be used to broaden the genetic base of clubroot resistance in *B*. *napus*. Introgression of important agronomic traits from *B*. *rapa* into *B*. *napus* through conventional breeding methods is possible [44–48], so resistance to clubroot could be transferred from *B*. *rapa* into *B*. *napus* through interspecific crosses. Conventional methods are increasingly supplemented with marker-assisted selection (MAS) using single nucleotide polymorphisms (SNP) and insertion / deletion (InDel) markers because of their high efficiency [49, 50]. Also, SNP markers occur abundantly in genomes and are amenable for use in high-throughput detection platforms [51].

Next generation sequencing (NGS) has ignited a revolution in life sciences. NGS has had a major impact on crop improvement, allowing the rapid development and application of genomics tools in plant breeding [52]. It enables the inexpensive and rapid discovery of DNA variants in many crops, including *Brassica* species [53, 54]. Another important technique, bulked segregant analysis (BSA), which consists of genotyping two bulks of individual plants with extreme phenotypes, is used to identify markers linked to disease resistance genes [55]. BSA and NGS were recently applied to mapping and marker development for disease resistance loci in several crops [56–59]. To minimize costs for NGS sequencing of complex genomes, BSA has also been coupled with transcriptional profiles from RNA sequencing (RNA-seq) data for mapping of QTLs and genes of interest [60–62].

More than 10 clubroot resistance genes and more than 20 QTLs have been identified in *Brassica* species and close relatives. However, no robust SNP markers for MAS are available. Recently, *Rcr1* was mapped to chromosome A03 of *B*. *rapa* based on resistance to pathotype 3 of *P*. *brassicae*, and differentially expressed genes associated with *Rcr1* were identified via RNA-seq [11]. The objectives of this study were to assess the efficacy of *Rcr1* against other pathotypes of interest to canola producers in Canada, and to use bulked segregant RNA-seq analysis to i) characterize genome-wide DNA variation; ii) examine DNA variation in the *Rcr1* target region and identify the most probable candidates of *Rcr1*; iii) develop SNP markers associated with *Rcr1*; and iv) validate SNP markers to select for *Rcr1* via MAS in canola (*B*. *napus*) breeding.

## Materials and Methods

### Development of F_2_ lines from a F_1_ segregating population and evaluation of the F_2_ lines for resistance to clubroot

Pak choy (*B*. *rapa* subsp. *chinensis*) cv. “Flower Nabana” (FN), a hybrid developed by Evergreen Y.H. Enterprises, Anaheim, CA, USA, is highly resistant to pathotype 3 of *P*. *brassicae*. *Rcr1* was identified and genetically mapped in 1587 plants from a F_1_ population that segregated for resistance (R) and susceptibility (S) [11]. This population was derived from a cross between FN, which is heterozygous at *Rcr1* locus, and canola line ACDC, which is a clubroot-susceptible *B*. *rapa* doubled haploid developed at the Saskatoon Research Centre of Agriculture and Agri-Food Canada. An additional 200 plants in the F_1_ population were self-pollinated, but most of the plants did not produce seed due to self-incompatibility in *B*. *rapa*. Seed was obtained from 38 plants, resulting in 38 F_2_ lines that could be evaluated for resistance to multiple pathotypes of *P*. *brassicae*. These lines were tested for resistance to pathotypes 2, 3, 5 and 6 under controlled conditions at the University of Guelph, ON, Canada. Plant seedlings were grown in soil-less mix in tall narrow plastic pots (164 mL conetainers, Stuewe & Sons Inc., Corvallis, OR, USA) and inoculated with 5 ml of resting spore suspension (1×10^6^ spores ml^−1^) at 10 days after seeding. Roots of 7-14 plants per line were assessed for clubroot severity at 6 weeks after inoculation using a standard 0 to 3 scale where: 0 = no clubbing; 1 = small clubs only; 2 = moderate clubs; and 3 = severe clubbing. A disease severity index (DSI) [63, 64] was calculated using the following formula:

$$DSI\;(\%)=\frac{\sum \;(rating\;class)\times (\#\;plants\;in\;rating\;class)}{total\;\#\;plants\;in\;treatment\times 3}\times 100$$

The highly susceptible Shanghai pak choy cv. “Mei Qing Choi” (Stokes Seeds, ON, Canada) was included as a susceptible control for each group to ensure that the inoculation was highly effective. Evaluation of the F_2_ lines and controls for resistance to the pathotypes of *P*. *brassicae* was replicated two times.

### RNA-seq and sequence alignment

At 15-days post-inoculation, root tissue from 9 R plants was combined to form the R bulk and from 9 S plants to form the S bulk; together, the two bulks comprised one biological replicate as described by Chu et al. 2014 [11]. In total, three replicates were assessed. The total RNA from each replicate was isolated using an RNeasy Plant Mini Kit (Qiagen; Toronto, ON, Canada) with oncolumn deoxyribonuclease (DNase) digestion using a Qiagen RNase-Free DNase set following the manufacturer’s instruction. The cDNA library was prepared using TruSeq RNA Sample Preparation Kits v2 (Illumina; San Diego, CA, USA). RNA-seq was carried out using a Illumina Hiseq 2500 platform (Plant Biotechnology Institute, National Research Council, Saskatoon, SK, Canada) and global gene expression by the RNA-seq was conducted using the *B*. *rapa* reference genome v1.2 (*B*. *rapa* subsp. *pekinensis* cv. “Chiifu”) [11]. In the current study, the short reads from the RNA-seq were aligned to the *B*. *rapa* reference genome v1.5, which was downloaded at http://brassicadb.org/brad/downloadOverview.php. The reference genome consists of 10 chromosomes and 40,357 scaffolds. The total lengths of chromosomes and scaffolds are about 258 million bases (Mb) and 27 Mb, equivalent to about 90% and 10% of the reference genome, respectively. To simplify downstream data analysis, only the 258 Mb chromosome sequences were used in the current study, so the short reads from the RNA-seq project were assembled into 10 chromosomes A01 to A10 of *B*. *rapa*. The program SeqMan NGen 11 (DNASTAR, Madison, WI, USA) was used for short read assembly. Two methods were used: 1) short reads from three biological replicates each of the R and S bulks were assembled into the reference genome, resulting in six assembly files—hereafter, this method is called the single sample assembly (SSA); 2) short reads from a pool of the three R bulks and a pool of the three S bulks were assembled into the reference genome, resulting in two assembly files—hereafter called the pooled sample assembly (PSA). Standard assembling and filtering parameters were used.

### Identification of variants

Discovery of variants (SNPs and InDels) in comparison with the DNA sequences in the *B*. *rapa* “Chiifu” [65] were performed using SeqMan Pro 11 (DNASTAR, Madison, WI, USA) with Q call ≥ 15 and depth ≥ 5. Analysis of variance and t-tests (LSD) were used to assess the variation in SNPs and InDels [66].

### Genotyping SNP markers

Selected SNPs identified in the target region were confirmed using the Kompetitive Allele Specific PCR (KASP) method (http://www.lgcgroup.com/) following the manufacture’s instruction. PCR reactions were performed using a StepOne Plus Real Time PCR System (Applied Biosystem, Mississauga, ON, Canada). FN8, a line derived from the *B*. *rapa* cv. FN and homozygous at the *Rcr1* locus, was included for this study.

To validate the SNP markers for MAS selection with *Rcr1*, DNA samples from 38 F_1_ plants from the cross between FN and the susceptible *B*. *rapa* line ACDC and 26 recombinants from the *B*. *rapa* population that was originally used to identify *Rcr1* [11] were assessed. The accuracy of the SNP markers for use in MAS was determined by testing the recombinants from a BC_1_ population derived from *B*. *napus* introgressed with *Rcr1* described by Chu et al, 2014 [11].

## Results

### Resistance to selected pathotypes

**Fig 1:**
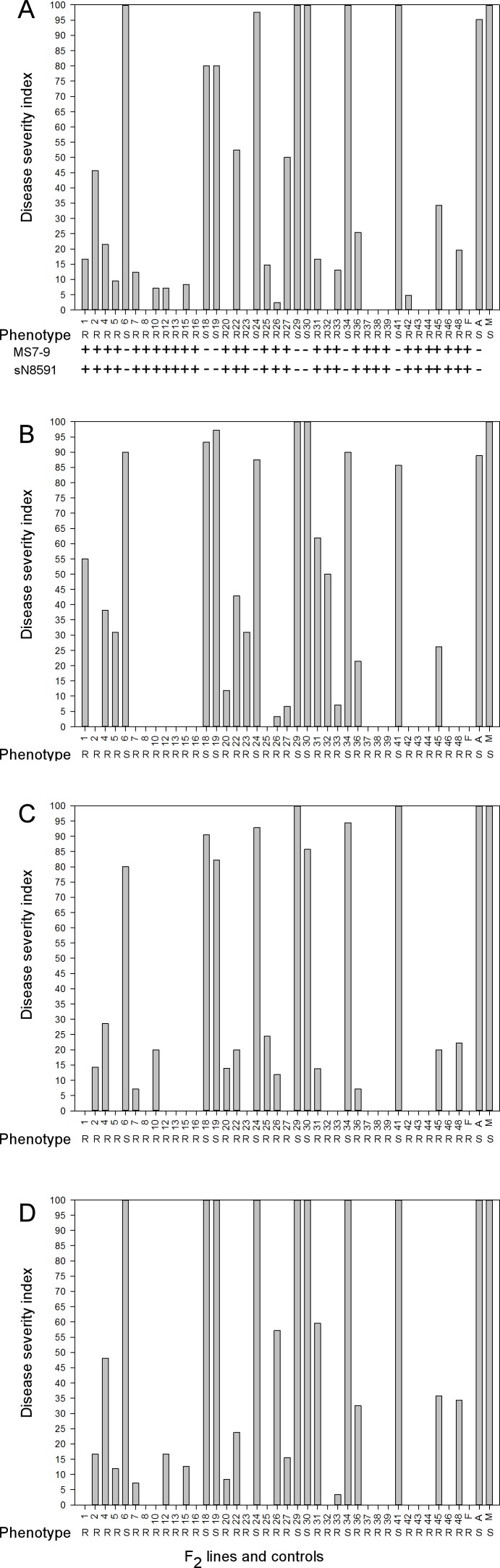
Evaluation of plants from 38 F_2_ lines for resistance to clubroot. A, Plants were inoculated with pathotype 3 and confirmed with molecular markers MS7-9 and sN8591 flanking *Rcr1* described by Chu et al [11]. “+”:heterozygous alleles, one allele from R parent and the other one from S parent, “-”:homozygous alleles from S parent; B, Inoculated with pathotype 2; C, Inoculated with pathotype 5; D, Inoculated with pathotype 6. Names of the lines indicate below X axis. Letters F, A, and M represent *B*. *rapa* cv. or breeding line FN, ACDC and “Mei Qing Choi”, respectively. Disease severity indexes (DSI) were calculated from clubroot severity ratings. R and S are the phenotypes assigned for each line based on DSIs. The experiments were replicated two times with similar results.

*Rcr1* was initially identified based on resistance to pathotype 3 (Williams’ differentials) [41] of *P*. *brassica*, the most prevalent pathotype identified in western Canada [11]. The resistant donor FN was found to be resistant to multiple pathotyes of *P*. *brassica* [43]. To determine if resistance to other pathotypes in the R donor cv. FN was also associated with *Rcr1*, we tested 38 F_2_ lines derived from the F_1_ segregating population for resistance to pathotypes 2, 3, 5 and 6. As expected, FN conferred resistance to pathotype 3 (0 DSI), but ACDC (95 DSI) and “Mei Qing Choi” (100 DSI) were highly susceptible (Fig 1). Eight of the 38 lines (F_2_-6, 18, 19, 24, 29, 30, 34 and 41) were highly susceptible to pathotype 3 (severity > 80 DSI) so their parental F_1_ plants likely did not carry *Rcr1*. Severity was low to moderate in the other 30 lines (0-52 DSI), which indicates that the parental F_1_ plants for these lines likely carried the *Rcr1* gene. To further confirm the F_1_ phenotypes determined by F_2_ lines, DNA samples from the original 38 F_1_ plants were assessed using two microsatellite markers, MS7-9 and sN8591, that flanked *Rcr1* [11]. The 30 F_1_ parental plants of the F_2_ lines with low to moderate DSI carried the allele associated with resistance; the 8 F_1_ plants with high DSIs in their F_2_ lines carried the allele associated with susceptibility (Fig 1), confirming the phenotypes in the 38 F_1_ plants determined by DSIs in the F_2_ lines.

The clubroot reaction of the parental lines and the F_2_ lines to pathotypes 2, 5 and 6 was also assessed. The parental cv. FN was highly resistance to pathotypes 2, 5 and 6, but ACDC and “Mei Qing Choi” were highly susceptible (Fig 1). The eight F_2_ lines that were susceptible to pathotype 3 were also highly susceptible to pathotypes 2, 5 and 6 (> 80 DSI). In contrast, clubroot severity was low to moderate (0-55 DSI) in the other 30 lines (Fig 1). Therefore, we conclude that resistance to pathotypes 2, 5 and 6 of *P*. *brassicae* was associated with resistance to pathotype 3 that was used for identification of *Rcr1* in the 38 lines.

### RNA-seq and sequence alignment

The average sequence counts and average accumulated sequence length were 125.9 million (M) and 11,093.8 Mb per R bulk, and 114.0 M and 10,005.8 Mb per S bulk using the SSA method (Table 1). This provided an average depth of coverage of the reference genome of 43 fold in R bulks and 39 fold in S bulks. The average sequence length assembled into the reference genome was 88 bases. More sequences were assembled into the longer chromosomes A03 and A09, while fewer sequences were assembled into the shorter chromosomes A04 and A10 (Table 1). The sequence counts assembled into the genome for each chromosome were highly correlated to chromosome length (r = 0.90) for both R and S bulks.

**Table 1:** Short reads assembled into chromosomes of the reference genome *B*. *rapa* “Chiifu” in single sample assembly (SSA) and pooled sample assembly (PSA)^a^.

Chromosome		Number of sequences (x 10^6^)				Accumulated length of sequences (bases x 10^6^)			
Number	Size (bases x 10^6^)	SSA		PSA		SSA		PSA	
		R	S	R	S	R	S	R	S
**A01**	26.9	12.9±1.4	11.8±3.1	35.9	33.2	1132.6±123.2	1029.6±271.6	3128.1	2881.3
**A02**	27.0	11.6±1.2	10.4±2.7	32.1	29.1	1019.1±105.3	907.8±235.9	2807.9	2539.0
**A03**	31.9	18.6±2.0	16.9±4.4	51.8	47.8	1627.9±173.8	1477.3±382.0	4516.7	4157.1
**A04**	19.3	8.6±0.9	7.8±2.0	23.5	21.5	764.2±80.9	685.5±176.2	2070.0	1883.6
**A05**	25.4	12.1±1.3	11.0±2.9	33.6	30.8	1067.5±116.3	963.7±251.3	2947.7	2695.6
**A06**	25.3	13.9±1.4	12.5±3.3	38.8	35.7	1230.0±128.9	1098.6±291.1	3423.7	3133.5
**A07**	25.9	12.0±1.3	10.9±2.8	33.9	31.1	1058.5±112.4	959.1±249.2	2977.3	2723.2
**A08**	20.9	9.9±1.1	9.0±2.4	27.7	25.6	864.6±95.5	785.6±208.5	2420.6	2225.4
**A09**	39.0	17.7±1.9	15.9±4.1	49.8	45.5	1561.7±169.9	1400.0±362.9	4369.2	3981.1
**A10**	16.4	8.7±0.9	7.9±2.1	24.6	22.7	767.7±82.5	698.6±185.8	2175.4	1996.6
**Total**	257.9	125.9±13.4	114.0±29.8	351.8	322.9	11093.8±1188.2	10005.8±2614.0	30836.5	28216.6

^a^ The data under SSA are mean ± SE.

Short reads from three R bulks and three S bulks were further assembled using the PSA method. As observed previously with the SSA method, more sequences were aligned into the longer chromosomes A03 and A09 and fewer sequences were aligned into the short chromosome A04 and A10 (Table 1). A total of 351.8 M sequences, 30,836.5 Mb in length, with coverage of 120 fold of the reference genome were assembled into *B*. *rapa* chromosomes from the pool of three R bulks, and 322.9 M sequences, 28,216.9 Mb in length, with 109 fold coverage were assembled from the pool of three S bulks (Table 1). About 50% of the aligned sequences were aligned to the top strand of the reference DNA and the other 50% to the bottom strand using the either the SSA or PSA methods (S1 Table).

### Identification of variants

**Fig 2:**
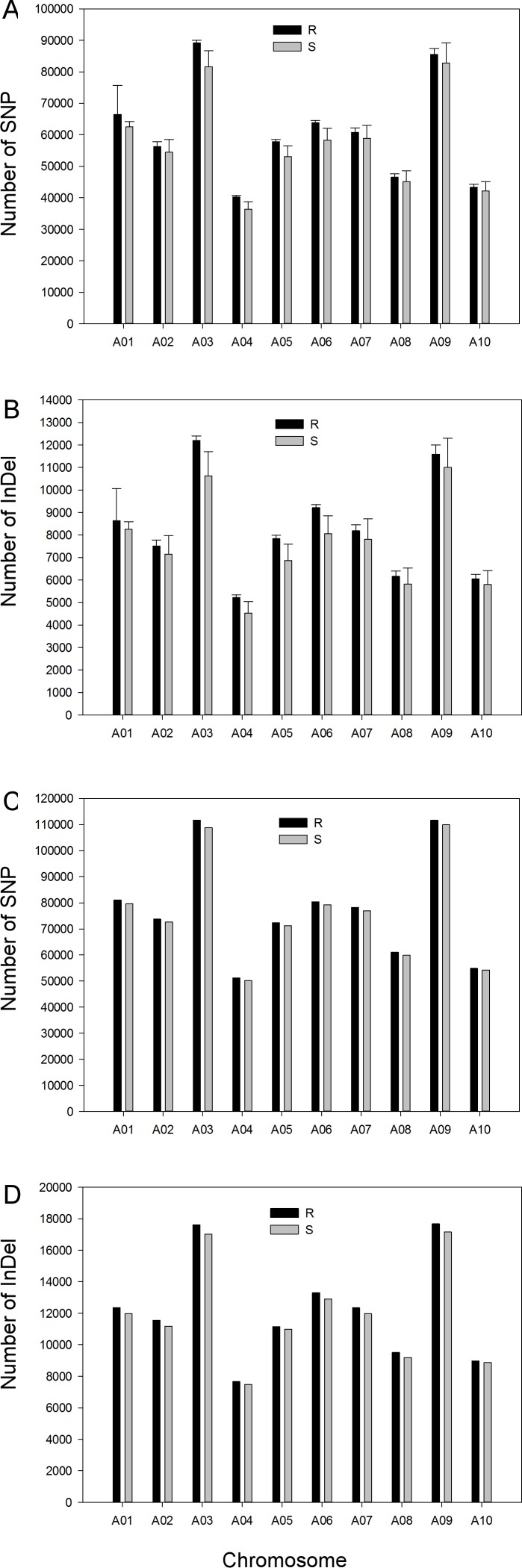
Numbers of variants (SNPs and InDels) identified in R and S bulks in comparing with the DNA sequence of the *B*. *rapa* cv. “Chiifu”. A, Number of SNPs by single sample assembly (SSA); B, Number of InDels by SSA; C, Number of SNPs by pooled sample assembly (PSA); D, Number of InDel by PSA.

Variants in both R and S samples were very frequent, with means of about 605.9 K SNPs and 82.2 K InDels per R bulk and 579.4 K SNPs and 76.3 K InDels per S bulk by using the SSA method. There was a strong positive correlation between the numbers of SNPs compared to InDels (r = 0.99 in both R and S bulks). The numbers of SNPs and InDels varied among the chromosomes (Fig 2), indicating that the longer chromosomes (A03, A09) carried more variants than the shorter chromosomes (A04, A10). However, the proportion of SNPs and InDels was similar among chromosomes, with about 88% and 12%, respectively, in both R and S bulks (S1 Fig).

The numbers of SNPs and InDels identified using the PSA method were higher than with the SSA method, with 776.2 K SNPs and 122.2 K InDels in the R bulk, and 762.8 K SNPs and 118.7 K InDels in the S bulk. As with the SSA method, there was a strong positive correlation between the numbers of SNPs and InDels with the correlation coefficient (r = 0.99) in either R or S bulks. The proportion of SNPs and InDels on each chromosome was 87% and 13% in both the R and S bulks (S1 Fig), which is a slightly lower proportion of SNP and higher proportion of InDel identified using the PSA method than the SSA method. As observed with the SSA method, the numbers of SNPs and InDels identified using PSA method (Fig 2) were higher on the longer chromosomes.

### Comparison of variants in R and S bulks

**Fig 3:**
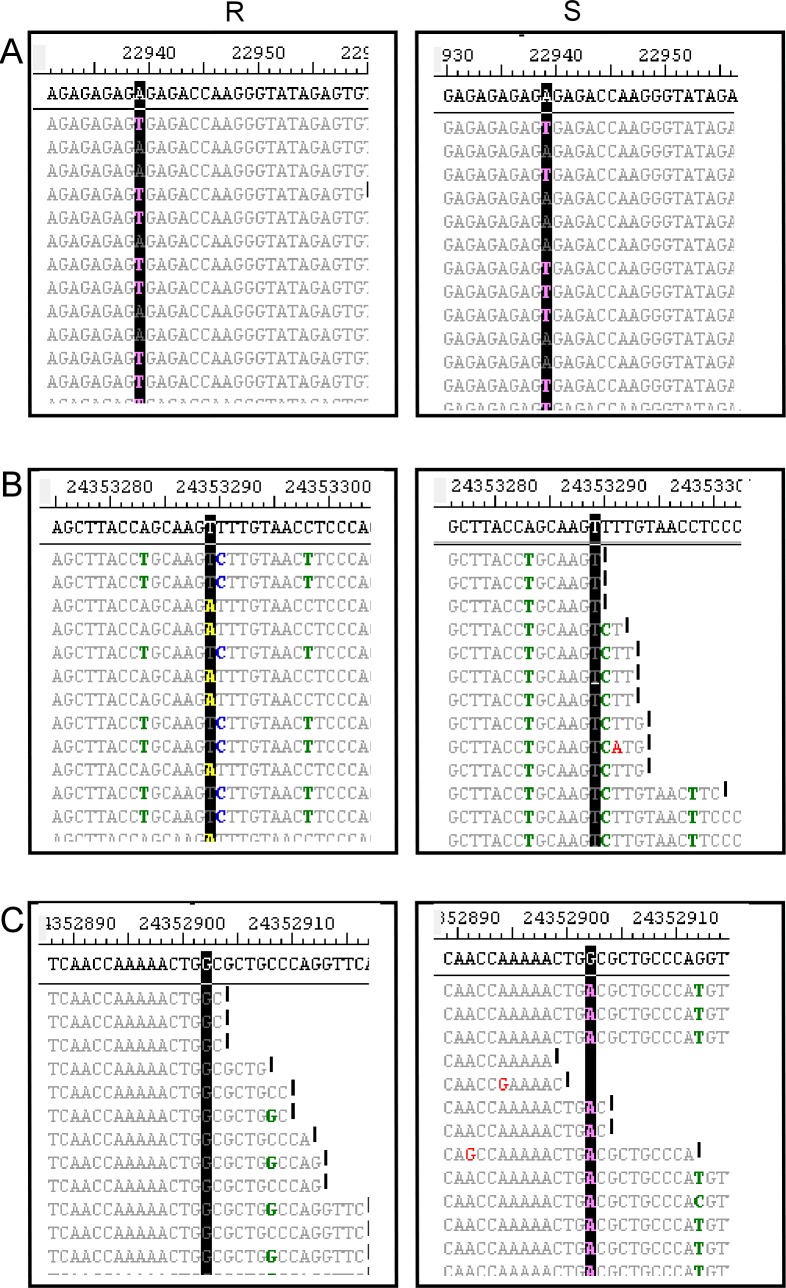
Examples of polymorphic and monomorphic variants on *B*. *rapa* chromosome A03 displayed by SeqMan Pro (DNASTAR, Madison, WI, USA). A, Monomorphic variant at the location 22939; SNP “T” in both R and S bulks; B, Polymorphic variant at the location 24353288; SNP “A” in R, but not in S; C, Polymorphic variant at the location 24352902; SNP “A” in S, but not in R. SNPs are highlighted in black. The numbers in the first row of each snapshot are the physical location on chromosome A03 in the *B*. *rapa* reference genome. The DNA sequences shown in the second rows are the reference genome sequences. The sequences below the reference genome are the short reads from either R or S bulks assembled into the reference genome.

**Fig 4:**
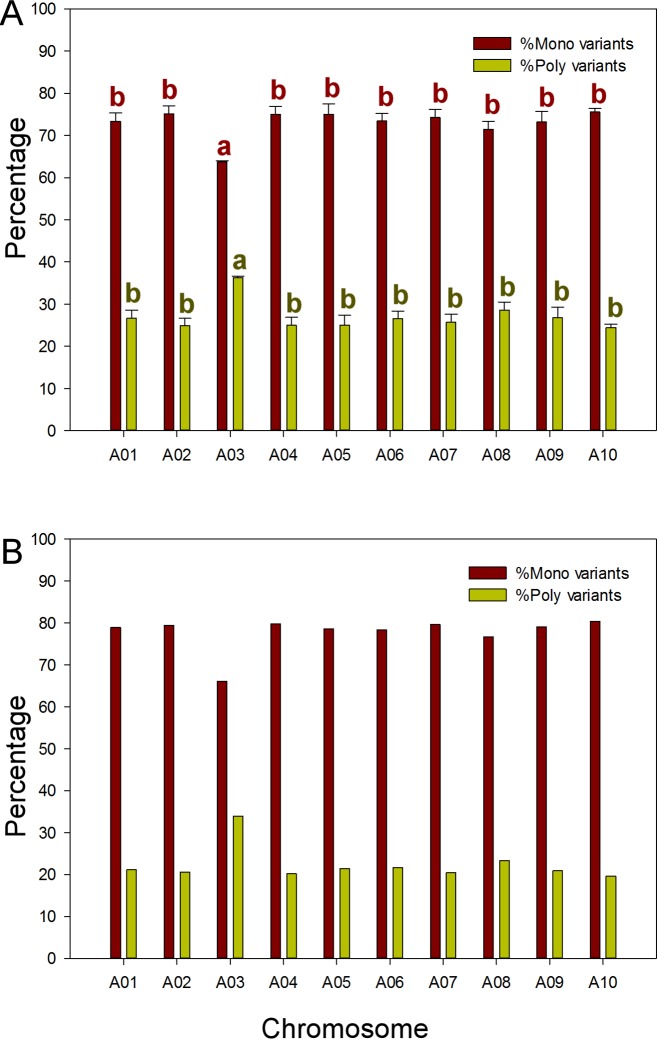
The proportion (%) of monomorphic and polymorphic variants on each chromosome. A, Using the single sample assembly method; and B, Using the pooled sample assembly method. Bars capped with the same letter do not differ at *P* ≤ 0.05.

Variants identified in the R and S samples could be the same (monomorphic, mono) or different (polymorphic, poly) (Fig 3). Mono variants comprised 73% (range 63.7-75.6%) of the variants identified across the *B*. *rapa* genome using the SSA method, and poly variants 27% (range 24.4%-36.3%). There were no differences among chromosomes in the proportion of mono and poly variants except for chromosome A03, where the clubroot resistance gene *Rcr1* is located, which carried more ploy variants and fewer mono variants (64%, *P* ≤ 0.05) than the other chromosomes (Fig 4).

With the PSA method, the proportion of mono variants was higher than for the SSA method for each chromosome (Fig 4). Overall, approximately 5% of the poly variants identified by SSA method were identified as mono using the PSA method. On chromosome A03, there were 66% mono variants and 34% poly variants, very similar to the results from SSA (Fig 4).

### Analysis of variants in the target region

A previous study reported that *Rcr1* was mapped in an interval of 0.28 centi-Morgan on *B*. *rapa* chromosome A03 and located in the 24.26-24.54 Mb of A03 [11]. In the current study, the target region was first analyzed using the SSA method. There were 35 genes annotated in the region, based on the reference genome v1.5 (Table 2). Three genes (*Bra038750*, *Bra019396* and *Bra019394*) did not show any expression and no short reads were assembled into the reference genome, so no variants could be identified. Short reads with sequence counts < 20 and number of variants < 1 was associated with 11 genes (*Bra038751*, *Bra038747*, *Bra019411*, *Bra019408*, *Bra019404*, *Bra019402*, *Bra019401*, *Bra019399*, *Bra019397*, *Bra019393* and *Bra019392*). More than 20 sequence counts with > 1 variants were associated with 21 genes in both R and S bulks (Table 2). Information on BLASTX (best hit) to *A*. *thaliana* and gene ontology annotation for the genes was obtained from http://brassicadb.org/brad/index.php (S2 Table). Four genes (*Bra019409*, *Bra019410*, *Bra019412* and *Bra019413*) encode TNL-class disease resistance proteins.

**Table 2:** Characterization of genes in the *Rcr1* target region based on location, number of sequences, and number of variants in the resistant (R) and susceptible (S) bulked samples.

Gene^a^	Location on A03	Number of sequences^b^		No. of variants^b^	
		R	S	R	S
Bra038758	24263024…24264157	193.3±4.5	118.3±3.9	1.7±0.1	0.3±0.1
Bra038757	24275881…24279414	289.0±5.7	248.3±9.8	28.3±0.6	19.0±0.9
Bra038756	24280484…24283179	1610±12.6	2170.0±125.4	18.3±0.1	10.0±0.2
Bra038755	24284960…24288356	287.7±6.2	481.0±23.4	14.0±0.5	7.0±0.4
Bra038754	24290155…24292149	5085.7 ±81.4	3473.0±156.9	33.7±0.2	26.0±0.5
Bra038753	24294019…24295276	2640±63.3	2456±100.3	19.7±0.2	17.7±0.1
Bra038751 (L)	24296257…24296659	4.3±0.2	3.7±0.1	0.0±0.0	0.3±0.1
Bra038750(N)	24303342…24304157	0.0±0.0	0.0±0.0	0.0±0.0	0.0±0.0
Bra038749	24306906…24310339	629.3±18.6	700.3±28.8	9.3±0.1	8.7±0.2
Bra038748	24323537…24327067	3393.3±84.2	2970.0±145.3	6.3±0.1	11.7±0.1
Bra038747 (L)	24339450…24340813	12.0±0.7	32.7±1.8	0.0±0.0	0.3±0.1
Bra019413	24350950…24353977	615.3±14.4	101.7±5.6	37.3±0.2	14.0±0.9
Bra019412	24370531…24371199	351.3±2.0	115.7±6.4	5.0±0.1	4.3±0.2
Bra019411 (L)	24372280…24372516	0.7±0.1	0.0±0.0	0.0±0.0	0.0±0.0
Bra019410	24373815…24379176	1156±18.4	493.7±25.6	94.7±0.5	45.3±1.8
Bra019409	24381590…24386315	548.0±10.5	710.7±36.3	71.3±0.5	57.7±1.4
Bra019408 (L)	24388976…24389449	1.3±0.1	1.3±0.2	0.0±0.0	0.0±0.0
Bra019407	24391878…24392811	745.3±20.3	951.0±47.4	3.3±0.1	2.7±0.1
Bra019406	24393210…24394793	4919.3±199.0	4304.3±212.0	46.7±0.1	30.0±0.0
Bra019405	24403694…24404722	35.7±1.9	73.3±4.6	0.7±0.1	2.0±0.1
Bra019404 (L)	24409283…24411164	10.0±0.4	6.7±0.4	0.0±0.0	0.3±0.1
Bra019403	24413728…24415552	70.3±1.2	2.0±0.2	16.3±0.3	0.3±0.1
Bra019402 (L)	24429989…24430549	1.3±0.2	2.0±0.0	0.0±0.0	0.0±0.0
Bra019401 (L)	24436080…24436250	0.7±0.2	0.0±0.0	0.0±0.0	0.0±0.0
Bra019400	24437662…24439848	32.3±4.8	325.0±25.0	0.7±0.2	6.0±0.5
Bra019399 (L)	24444666…24444989	0.0±0.0	1.0±0.3	0.0±0.0	0.0±0.0
Bra019398	24454780…24457677	45.3±2.3	22.3±2.0	3.7±0.4	0.3±0.1
Bra019397 (L)	24480924…24483948	4.0±1.0	2.0±0.0	0.0±0.0	0.0±0.0
Bra019396(N)	24501547…24502262	0.0±0.0	0.0±0.0	0.0±0.0	0.0±0.0
Bra019395	24510450…24512273	1327.0±43.9	1127±99.0	11.3±0.1	2.3±0.1
Bra019394(N)	24517143…24519546	0.0±0.0	0.0±0.0	0.0±0.0	0.0±0.0
Bra019393 (L)	24520116…24521078	20.0±0.6	1.0±0.4	0.7±0.3	0.0±0.0
Bra019392 (L)	24525560…24526147	12.7±0.9	10.7±2.3	0.0±0.0	0.0±0.0
Bra019391	24529596…24530189	57.3±8.0	83.7±10.7	1.0±0.3	4.7±0.7
Bra019390	24534962…24537043	7449.0±1215.0	6775.0±1473.0	32.0±1.2.0	24.7±2.0.0

^a^(L) = Little expression; (N) = No expression

^b^Mean ± SE.

The numbers of poly variants between the R and S bulks among the genes in the coding regions was assessed because the poly variants represent differences in the DNA sequences between the R and S bulks. The numbers of poly variants differed significantly among the 21 genes (Table 3).Three TNL genes (*Bra019409*, *Bra019410*, and *Bra019413*) had the most poly variants, with means of 81.7, 75.0 and 34.7 poly variants per gene, respectively, followed by four genes (*Bra019406*, *Bra038754*, *Bra019390* and *Bra038757*) with means of 33.3, 31.3, 29.0 and 24.0. These seven genes exhibited more poly variants than the other 14 genes (*P* ≤ 0.05). The fourth TNL-class gene (*Bra019412*), with mean of 4.0 poly variants, was one of the 14 genes that showed few polymorphic variants (Table 3).

**Table 3:** Number of polymorphic variants identified in each gene located in the *Rcr1* interval in the simple sequence assembly (SSA) and pooled sequence assembly (PSA) analysis.

Gene	No. of variants (SSA) ^a^	No of variants (PSA)
**Bra019409**	81.7±1.2 a	115
**Bra019410**	75.0±0.9 b	85
**Bra019413**	34.7±0.4 c	52
**Bra019406**	33.3±0.1 cd	37
**Bra038754**	31.3±0.5 cd	39
**Bra019390**	29.0±0.8 de	38
**Bra038757**	24.0±0.3 e	31
**Bra019403**	16.7±0.5 f	18
**Bra038753**	14.3±0.3 fg	20
**Bra038755**	13.0±0.6 fgh	17
**Bra038756**	11.3±0.2 gh	9
**Bra019395**	9.7±0.3 ghi	12
**Bra038748**	8.0±0.1 hij	11
**Bra019391**	5.7±0.8 ijk	10
**Bra038749**	5.3±0.2 ijk	5
**Bra019400**	5.3±0.9 ijk	4
**Bra019412**	4.0±0.4 jk	4
**Bra019398**	4.0±0.5 jk	9
**Bra038758**	2.0±0.1 k	3
**Bra019407**	1.7±0.1 k	3
**Bra019405**	1.3±0.1 k	2

^a^ Mean (± SE) followed by the same letter do not differ at *P* ≤ 0.05.

The PSA method identified more poly variants among the 21 genes in the target region than the SSA method (Table 3). For both methods, three TNL genes (*Bra019409*, *Bra019410*, and *Bra019413*) consistently carried the highest number of poly variants among the 21 genes.

### Analysis of variant types in the TNL genes

**Fig 5:**
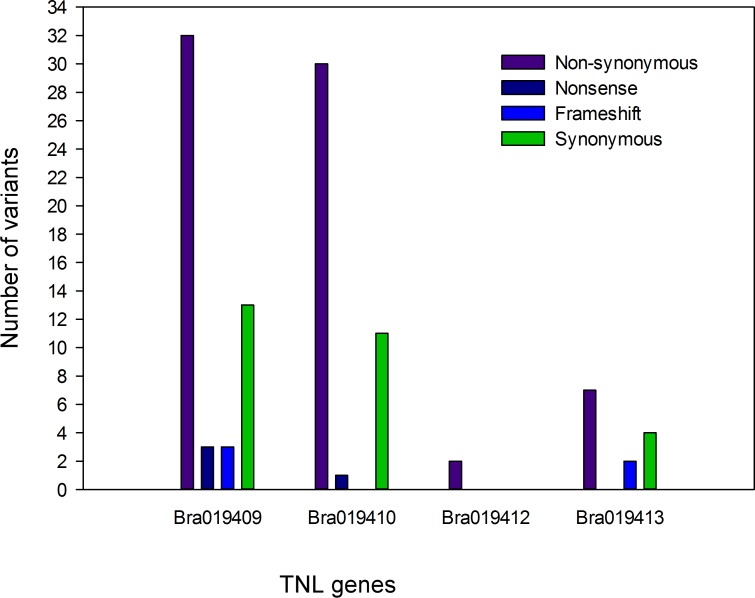
Number of unique poly variants in the four TIR-NBS-LRR genes from the resistant bulked samples.

The poly variants that uniquely occurred in the R bulks but not in the S bulks (Fig 3) with depth > 5 in both samples were further assessed using the PSA method. A total of 108 poly variants were identified from the coding sequences in the four TNL genes (S3 Table). SeqMan Pro software was used to sort the 108 variants that affect amino acid sequences into four groups: non-synonymous, nonsense, frameshift and synonymous variants. Non-synonymous variants occurred in each of the TNL genes, but *Bra019409* and *Bra019410* carried many more non-synonymous variants than the other two TNL genes (Fig 5). Three nonsense variants were identified in *Bra019409* and one in *Bra019410*, but none were identified in the other two genes. Frameshift variants caused by InDels were found in *Bra019409* and *Bra019413*, and synonymous variants occurred in each of the genes except *Bra019412* (Fig 5). Overall, *Bra019409* and *Bra019410* carried higher number of poly variants that uniquely occurred in the R samples and could affect the amino acid sequence in their proteins.

### Genotyping SNP markers identified in the *Rcr1* region

**Fig 6:**
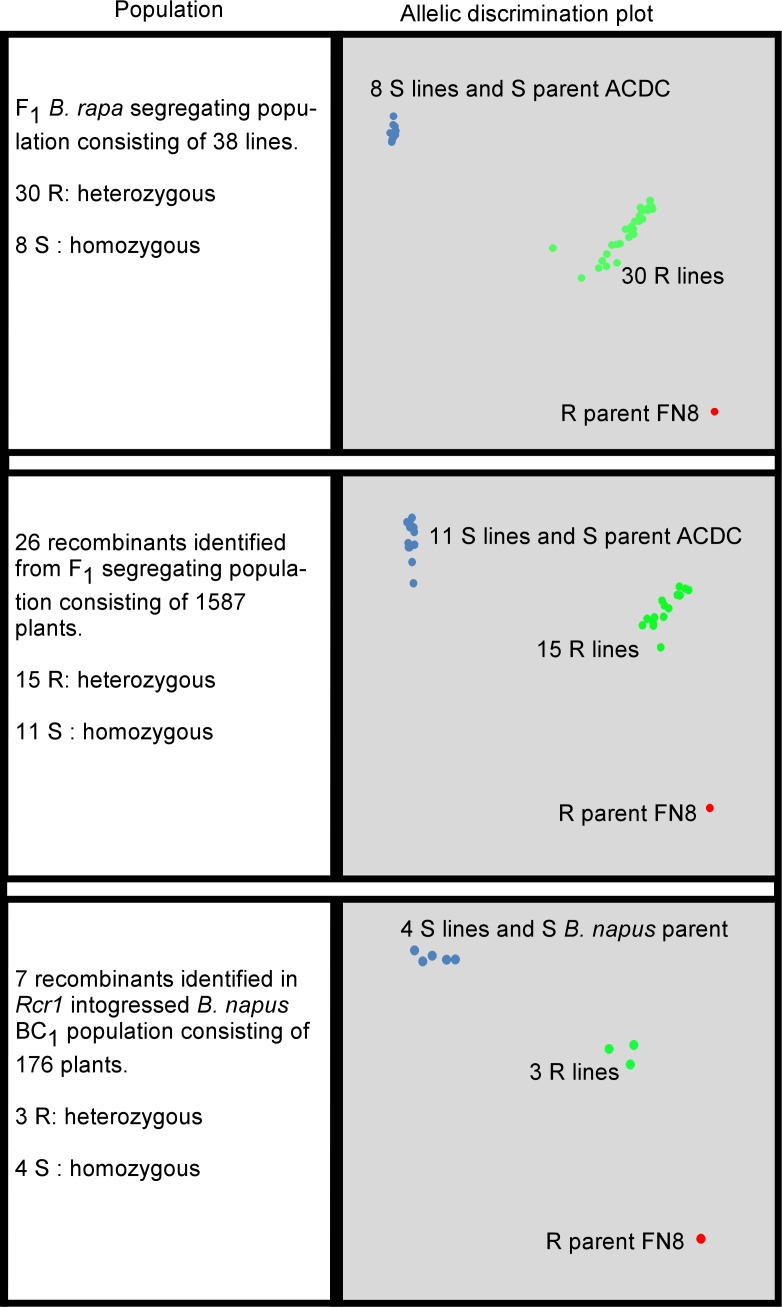
Allelic discrimination plots show the genotypes of three populations based on SNP marker, SNP_A03_12, using the KASP method. ACDC and FN8 are homozygous susceptible and resistant lines, respectively.

The DNA sequences flanking 11 SNPs in the four TNL genes (S3 Table) and three SNPs (SNP_A03_18, 19, and 32) in the gene *Bra019406* that was one of genes harboring more poly variants (Table 3) were obtained from the *B*. *rapa* reference genome (S4 Table). These SNP markers, which spanned about 42 Kb in the target region (Table 4), were genotyped using the KASP method. As expected, each SNP marker was polymorphic between the R parental line FN8 and the S parental line ACDC (Fig 6), which confirmed that a SNP was present at each locus. In an allelic discrimination plot, PCR products from the FN8 (R) were usually close to the right bottom and ACDC (S) to the left top (Fig 6). This indicates that they carried homozygous alleles that might be associated with R and S, respectively. In contrast, when DNA samples from the 38 F_1_ plants (Fig 1) were analyzed using the 14 SNP markers, the PCR products from 30 R plants were in the middle (Fig 6). This indicates that these plants were heterozygous at this locus (Table 4), with one allele from the R parent and the other one from the S parent. PCR products from the eight S plants were close to the right top (Fig 6), where the PCR product from the susceptible line ACDC was located, indicating that they carried homozygous alleles from the S parent (Table 4). These results confirm that the 14 SNP markers identified by RNA-seq were completely associated with *Rcr1* on chromosome A03 in this population.

**Table 4:** Association between disease reaction phenotype and SNP markers in the F_1_ segregating populations.

SNP marker	Gene	A03 location^a^	Phenotype^b^	Number of plants with marker^c^	
				+	-
**SNP_A03_11**	Bra019413	24352616	R	30	0
			S	0	8
**SNP_A03_104**	Bra019413	24352908	R	30	0
			S	0	8
**SNP_A03_08**	Bra019413	24353289	R	30	0
			S	0	8
**SNP_A03_09**	Bra019412	24371044	R	30	0
			S	0	8
**SNP_A03_102**	Bra019410	24375018	R	30	0
			S	0	8
**SNP_A03_12**	Bra019410	24375572	R	30	0
			S	0	8
**SNP_A03_13**	Bra019410	24375979	R	30	0
			S	0	8
**SNP_A03_14**	Bra019410	24376507	R	30	0
			S	0	8
**SNP_A03_103**	Bra019410	24378671	R	30	0
			S	0	8
**SNP_A03_16**	Bra019409	24382012	R	30	0
			S	0	8
**SNP_A03_101**	Bra019409	24382587	R	30	0
			S	0	8
**SNP_A03_18**	Bra019406	24393429	R	30	0
			S	0	8
**SNP_A03_19**	Bra019406	24393825	R	30	0
			S	0	8
**SNP_A03_32**	Bra019406	24394435	R	30	0
			S	0	8

^a^Physical location on chromosome A03 based on *Brassica rapa* reference genome v1.5.

^b^Phenotypes in 38 F_1_ plants were determined in F_2_, as shown in Fig 1.

^c^ Genotyping SNP markers in the *Rcr1* target region using KASP method: “+” allele from the resistant parent FN; “-” allele from the susceptible parent ACDC.

**Fig 7:**
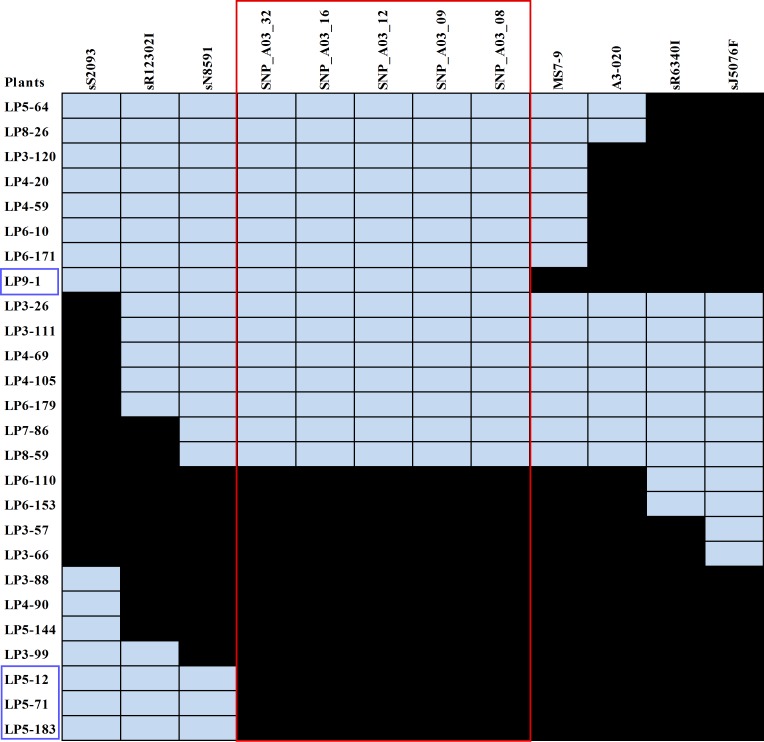
Analysis of the recombinants selected from the F_1_ population derived from the cross ACDC x FN. Phenotypes (R for resistant, S for susceptible) on the right, molecular markers (sS2093, sR12302I, sN8591, MS7-9, A3-020, sR6340I and sJ5076F) on the top were described previously [11]. The recombinants were analyzed with five SNP markers (SNP_A03_08, 09, 12, 16 and 32) residing in the genes *Bra019413*, *Bra019412*, *Bra019410*, *Bra019409* and *Bra019406* respectively using the KASP method. They are highlighted in the red rectangle. PCR products amplified from R alleles are denoted in light grey and those from S alleles in black. The four recombinants in blue rectangles were identified with the closest flanking markers sN8591 and MS7-9 [11].

To determine if the SNP markers were located in the target region, we analyzed 26 recombinants, including LP9-1, LP5-12, LP5-71 and LP5-183 that were identified by the closest flanking markers, MS7-9 and sN8591 from 1587 F_1_ plants with five SNP markers (SNP_A03_08, 09, 12, 16, and 32) that reside in the genes *Bra019413*, *Bra019412*, *Bra019410*, *Bra019409* and *Bra019406* respectively (Fig 6). Each SNP marker co-segregated with phenotypes and no recombination event was found in these plants (Fig 7). This indicates that the SNP markers were completely associated with the clubroot resistance gene *Rcr1*.

### Validation of SNP markers for MAS of *Rcr1* in canola breeding

To assess the potential use of the SNP markers in MAS for introgression of *Rcr1* into canola, DNA samples were assessed from seven recombinants in the BC_1_ populations consisting of 176 plants, which were previously used for validating *Rcr1* linked to microsatellite markers for MAS [11], with the 14 SNP markers (Table 4). Five SNP markers (SNP_A03_16, 18, 19, 32, 103) were polymorphic in the *B*. *rapa* population; however, they were monomorphic in the *B*. *napus* population. Polymorphism was identified in the rest of 9 SNP markers (Table 5). The SNP alleles from the R parent occurred in 3 R plants (BN-4, 27, and 58) and the alleles from the S parent occurred in 4 S plants (BN-1, 86, 103, and 113) (Fig 6; Table 5). The accuracy for MAS in the BC_1_ population using the 9 KASP SNP markers was 100%, and the SNP markers completely co-segregated with the phenotypes.

**Table 5:** Validation of SNP markers for detecting *Rcr1* in BC_1_ populations derived from a cross of FN x *Brassica napus* described by Chu et al [11] ^a^.

Plant	FN	*B*.* napus*	BN-01	BN-04	BN-27	BN-58	BN-86	BN-103	BN-113
Phenotype	R	S	S	R	R	R	S	S	S
**SNP_A03_11**	+	-	-	+	+	+	-	-	-
**SNP_A03_104**	+	-	-	+	+	+	-	-	-
**SNP_A03_08**	+	-	-	+	+	+	-	-	-
**SNP_A03_09**	+	-	-	+	+	+	-	-	-
**SNP_A03_102**	+	-	-	+	+	+	-	-	-
**SNP_A03_12**	+	-	-	+	+	+	-	-	-
**SNP_A03_13**	+	-	-	+	+	+	-	-	-
**SNP_A03_14**	+	-	-	+	+	+	-	-	-
**SNP_A03_101**	+	-	-	+	+	+	-	-	-

^a^ “+” allele from the R parent FN; “-” from S parent *B*. *napus*.

## Discussion

More than 10 clubroot resistance genes including *CRa*, *CRb*, *CRc*, *CRk*, *Crr1*, *Crr2*, *Crr3* and *Crr4* were identified previously in vegetable cultivars of *B*. *rapa* [9, 10, 12–23]. These genes were identified based on the reaction to the *P*. *brassicae* pathotypes prevalent in Japan, Korea and China, which would likely be classed into pathotypes 2 or 4 based on Williams’ differential hosts [41]. At least five pathotypes of *P*. *brassicae* (2, 3, 5, 6 and 8, again based on Williams’ differentials) occur in Canada. Pathotype 3 is the most prevalent on canola [67, 68].

*Rcr1* was previously mapped on chromosome A03 based on resistance to pathotype 3 [11], but the donor cv. FN was resistant to each of the pathotypes previously identified in Canada [43]. To determine if *Rcr1* confers resistance against the range of Canadian pathotypes, a F_2_ population consisting of 38 lines was developed. The F_2_ lines were equivalent to self-pollinated BC_1_ populations (BC_1_S_1_). There were two genotypes “Rr”, heterozygous at *Rcr1* locus, which should be resistant and “rr”, homozygous susceptible alleles, which should be susceptible phenotype in F_1_. Theoretically, segregation for resistance and susceptibility from “Rr” plants in the F_2_ lines is 3:1 and that from “rr” should be 0:1. Ideally, X^2^ analysis would have been performed. Unfortunately, the small number of plants available for evaluation of plant resistance in this study made X^2^ analysis impractical. We speculated that the eight F_2_ lines with high severity were from “rr” F_1_ plants and the 30 F_2_ lines with low to moderate severity were from “Rr” F_1_ plants. Similar results were obtained by repeating the experiments. In addition, the results were confirmed by analysis of the molecular markers (Fig 1, Table 4). Thus, the genotypes of the F_1_ plants determined by the phenotypes in F_2_ lines in this study were reliable, even though 7 to 14 plants each line for each pathotype were tested per replicate. In some of the F_2_ lines that were derived from “Rr” F_1_ plants, their DSIs were as low as zero. This could be caused by segregating distortion so no “rr” plants appeared in the small populations. In addition, disease escapes from “rr” plants could contribute to the zero DSIs. Resistance to pathotypes 2, 3, 5 and 6 was completely linked in each of the 38 lines. However, variation was usually found among DSIs of the same F_2_ lines responding to different pathotypes. The reason for the variation could be that the proportions of the genotypes (RR, Rr and rr) for those “Rr” derived F_2_ lines against each pathotype were not exactly same. It also could be caused by some escapes from “rr” plants especially for those “rr” derived F_2_ lines. The original intent was to assess 200 F_1_ plants in this study, but only these 38 plants produced sufficient seed in the F_2_ generation for assessment. Resistance to pathotype 8 was also associated with resistance to pathotype 3 in one iteration of the assessment (data not show), but there was not sufficient seed to repeat that portion of the study. Resistance to this range of pathotypes indicates that *Rcr1* could confer broad spectrum resistance to many of the pathotypes of *P*. *brassicae* that are present in Canada. Another possibility is that the resistance to this range of pathotypes could be controlled by linked genes. Clearly, this still needs to be determined.

RNA-seq generates a wealth of short DNA sequence reads from random places in the transcriptome. Transcriptome analysis and identification of SNPs based on RNA-seq have been carried out recent years in many organisms, including *Brassicas* such as *B*. *napus* [53, 69, 70] and *B*. *rapa* [11, 54]. The current study focused on characterization of variants in the *B*. *rapa* population that carried *Rcr1*, and identified the most probable candidates for *Rcr1* based on DNA variants and development of SNP markers tightly linked to the gene for MAS. Also, SNP variants were found to be the most common DNA sequence variation, accounting for over 80% of the *B*. *rapa* transcriptome in the current study. Similar results were reported in rice, with an average of 99,955 putative SNPs and 14,617 putative InDels [71] and the human genome, where SNPs made up about 3 million of the 4 million DNA sequence [72]. Recently, RNA-seq of human airway epithelial cells revealed that over 90% of variants were SNPs [73].

A wealth of SNPs and InDels were identified in the *B*. *rapa* genome using both the SSA and PSA approaches, which generally produced similar results. The SSA method, in which three biological replicates of R and S bulks were assessed, provided estimates of variance that could be used to assess the statistical significance of variation of SNPs and InDels. This approach is very useful for identifying differentially expressed genes through RNA-seq. In contrast, the PSA approach, which merged the data across biological replicates, is much less amenable to statistical analysis. However, it produced a greater depth of sequencing data, providing more reliable results in discovery of variants. Therefore, this approach would be preferred in future SNP discovery through bulked segregant RNA sequencing.

In both R and S samples, variants from the reference genome DNA sequences in *B*. *rapa* Chinese cabbage “Chiifu” v1.5 [65] were very frequent. One factor that likely contributed to this high frequency of variants was that the parental lines were pak choy and canola, rather than Chinese cabbage. Deep sequencing, with coverage of 120 fold of the reference genome in R and 109 fold in S samples, could also have contributed to the wealth of variants in the current study. More than 70% of the variants between R and S bulks were monomorphic in all chromosomes except A03, where a significantly higher percentage of polymorphic variants were present. The high frequency of poly variants could indicate that there was a higher level of difference between the R and S bulks in *Rcr1* and its surrounding region than in other parts of genome.

*Rcr1* was fine mapped in an interval of 0.28 centi-Morgan, flanking by microsatellite markers MS7-9 and sN8591 on *B*. *rapa* chromosome A03 [11]. In this study, we identified more than 400 variants (Table 3) in the target region. Fourteen SNP markers that reside in five genes (*Bra019406*, *Bra019409*, *Bra019410*, *Bra019412* and *Bra019413*) in the target region were genotyped using KASP method. Each of the markers co-segregated with *Rcr1*, indicating that they were completely linked to *Rcr1* and closer to *Rcr1* than the markers previously described by Chu et al 2014 [11]. However, it was not possible to define the *Rcr1* location further with the recombinants in the mapping population, because no recombination events could be identified in the set of recombinants. Therefore, it was essential to obtain accurate SNP and Indel profile on the genes of interest through analysis of RNA-seq data. Four of the five genes identified encode proteins related to disease resistance that belong to TNL families. Non-synonymous, nonsense, frameshift and synonymous variants were identified in the TNL genes. It is unlikely that the genes *Bra019412* and *Bra019413* are the candidates for *Rcr1;* although they showed differential expression between R and S bulks [11], few poly variants, especially variants that cause changes in amino acid sequence, were identified in these genes. *Bra019409* and *Bra019410* carried higher number of variants that could cause changes in amino acid sequence and higher number of synonymous variants, previously considered as 'silent', but which can cause changes in protein expression, conformation and function [74]. Therefore, it is more likely that either of these genes is a candidate for *Rcr1*. It is also possible that a combination of the TNL genes is required for the expression of *Rcr1* resistance. *Rcr1* was mapped in the same interval as one of the cloned clubroot resistance gene *CRa* [11]. However, it was not illustrated how *CRa* was related to the TNL genes in the target region [38]. The complete *CRa* coding sequence is 4223 bp (GenBank: AB751517.1). We searched the *B*. *rapa* genome using the 4223 bp sequence at http://brassicadb.org/brad/blastPage.php and found that *CRa* was homologous to the four TNL genes *Bra019410*, *Bra019412*, *Bra019409* and *Bra019413* with score and E value at 1905, 0.0; 823, 0.0; 739, 0.0 and 504, e-^141^ respectively. This indicates that *CRa* is a gene homologous to the TNL genes especially *Bra019410*, but obviously different from any of the TNLs. Identifying the TNL gene that corresponds with *Rcr1* and relationship of *Rcr1* with *CRa* will be addressed after *Rcr1* has been cloned.

Breeding for clubroot resistance in canola at many locations in Canada is severely constrained because the pathogen, or the pathotype of interest, does not occur at or near the site of established breeding institutions. Use of highly specific markers in MAS could be to accurately assess the reaction of lines under controlled (i.e. indoor) conditions. A number of techniques are available for genotyping SNPs. NGS is an emerging method of SNP genotyping that is being increasingly adopted for discovery applications. However, this method can be expensive and time-consuming in terms of informatics needs, and currently generates datasets with a large proportion of missing data [75]. KASP offers cost-effective and scalable flexibility in applications [76]. In total, 14 robust SNP markers were confirmed that were completely associated with *Rcr1*, based on testing the recombinants identified from 1587 plants. Most of the SNPs proved to be very reliable markers in use for introgression of *Rcr1* into elite canola breeding lines. Furthermore, MAS with the polymorphic SNP markers was confirmed to have detected *Rcr1* in the BC_1_ population described by Chu et al [11] with 100% accuracy in this study. Therefore, we conclude that these SNP markers could provide an effective and robust basis for introgression of *Rcr1* into canola using MAS.

## Supporting Information

S1 Fig Percentages of SNPs and InDels on each chromosome. A, R bulks by SSA; B, S bulks by SSA; C. R bulks by PSA; D. S bulks by PSA.
(PDF)

S1 Table Millions of short sequences from samples of resistant (R) and susceptible (S) plants, assembled into top and bottom strands of chromosomes of the reference genome *B*. *rapa* “Chiifu” using single simple assembly (SSA) and pooled simple assembly (PSA). (DOCX)

S2 Table Best hit to *Arabidopsis thaliana* genome and gene ontology annotation for the genes in the *Rcr1* target region. (DOCX)

S3 Table Polymorphic variants identified from R bulks in the TIR-NBS-LRR genes. (DOCX)

S4 Table SNPs and their flanking sequences. (DOCX)
