# Supplementary figures and images for: Identification of Genome-Wide Variants and Discovery of Variants Associated with Brassica rapa Clubroot Resistance Gene Rcr1 through Bulked Segregant RNA Sequencing
Source: PLoS One. 2016 Apr 14;11(4):e0153218. doi: 10.1371/journal.pone.0153218 (PMC4831815; doi:10.1371/journal.pone.0153218)

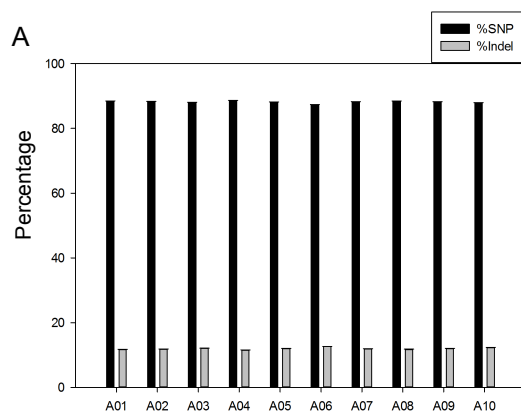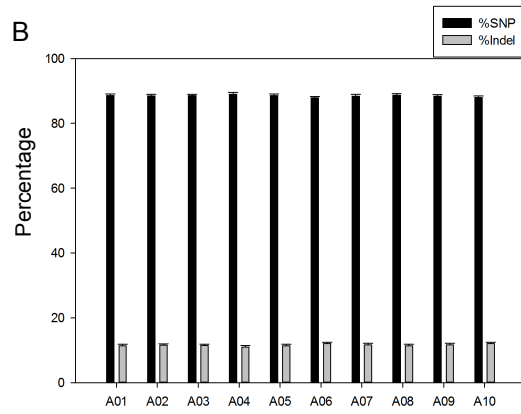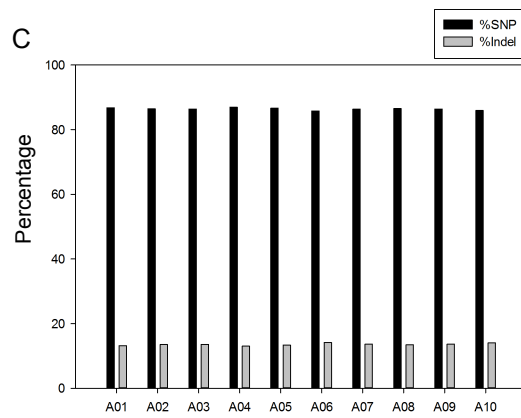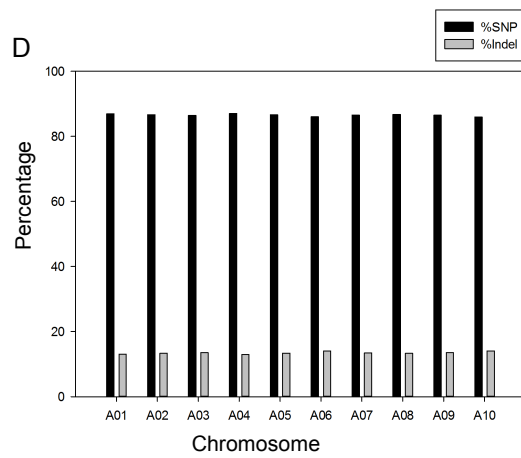

Supplement: S1 Fig — A, R bulks by SSA; B, S bulks by SSA; C. R bulks by PSA; D. S bulks by PSA. (PDF) [file pone.0153218.s001.pdf]
