# Supplementary material for: Identification of Genome-Wide Variants and Discovery of Variants Associated with Brassica rapa Clubroot Resistance Gene Rcr1 through Bulked Segregant RNA Sequencing
Source: PLoS One. 2016 Apr 14;11(4):e0153218. doi: 10.1371/journal.pone.0153218 (PMC4831815; doi:10.1371/journal.pone.0153218)
Supplement: S1 Table — (DOCX) [file pone.0153218.s002.docx]

**S1Table. Millions of short sequences from samples of resistant (R) and susceptible (S) plants, assembled into top and bottom strands of chromosomes of the reference genome *B. rapa* “Chiifu” using single simple assembly (SSA) and pooled simple assembly (PSA)^a^.**

| **Chr** | **SSA** | | | | **PSA** | | | |
| --- | --- | --- | --- | --- | --- | --- | --- | --- |
|  | **R** | | **S** | | **R** | | **S** | |
|  | **Top** | **Bottom** | **Top** | **Bottom** | **Top** | **Bottom** | **Top** | **Bottom** |
| **A01** | 6.5±0.7 | 6.5±0.7 | 5.9±1.6 | 5.9±1.6 | 18.1 | 17.8 | 16.7 | 16.5 |
| **A02** | 5.8±0.6 | 6.0±0.6 | 5.2±1.4 | 5.2±1.3 | 16.2 | 15.9 | 14.7 | 14.4 |
| **A03** | 8.2±1.6 | 8.1±1.6 | 8.5±2.2 | 8.4±2.2 | 26.0 | 25.7 | 24.0 | 23.7 |
| **A04** | 6.0±1.6 | 5.9±1.6 | 3.9±1.0 | 3.9±1.0 | 11.8 | 11.7 | 10.8 | 10.7 |
| **A05** | 5.5±0.9 | 5.5±0.9 | 5.5±1.4 | 5.4±1.4 | 17.0 | 16.7 | 15.5 | 15.3 |
| **A06** | 6.7±0.8 | 6.6±0.8 | 6.3±1.7 | 6.2±1.6 | 19.5 | 19.3 | 18.0 | 17.7 |
| **A07** | 6.3±0.7 | 6.3±0.7 | 5.5±1.4 | 5.4±1.4 | 17.0 | 16.8 | 15.6 | 15.4 |
| **A08** | 5.3±0.6 | 5.3±0.6 | 4.5±1.2 | 4.5±1.2 | 13.9 | 13.8 | 12.8 | 12.7 |
| **A09** | 7.6±1.7 | 7.5±1.7 | 8.0±2.1 | 7.9±2.1 | 25.0 | 24.8 | 22.9 | 22.7 |
| **A10** | 5.8±1.5 | 5.8±1.5 | 4.0±1.1 | 3.9±1.0 | 12.4 | 12.2 | 11.4 | 11.3 |
| **Total** | 63.6±6.6 | 63.5±6.7 | 57.2±15.0 | 56.8±14.9 | 177.0 | 174.8 | 162.5 | 160.3 |

^a^ The data under SSA are mean ± SE.
