# Supplementary material for: Identification of Genome-Wide Variants and Discovery of Variants Associated with Brassica rapa Clubroot Resistance Gene Rcr1 through Bulked Segregant RNA Sequencing
Source: PLoS One. 2016 Apr 14;11(4):e0153218. doi: 10.1371/journal.pone.0153218 (PMC4831815; doi:10.1371/journal.pone.0153218)
Supplement: S2 Table — (DOCX) [file pone.0153218.s003.docx]

**S2 Table. Best hit to *Arabidopsis thaliana* genome and gene ontology annotation for the genes in the *Rcr1* target region^a^**

| **Gene name** | ***A. thaliana* annotation** | **Molecular function** | **Biological process** | **Cellular component** |
| --- | --- | --- | --- | --- |
| **Bra038758** | Ric10 (rop interactive crib motif-containing protein 10) | - | - | - |
| **Bra038757** | Atml1; atml1 (meristem layer 1) | Sequence-specific DNA binding; transcription regulator activity; transcription factor activity | Regulation of transcription, DNA-dependent | nucleus |
| **Bra038756** | Bglu47; Bglu47 (Beta-glucosidase 47) | Hydrolase activity, hydrolyzing O-glycosyl compounds | Carbohydrate metabolic process | - |
| **Bra038755** | Bglu47; Bglu47 (Beta-glucosidase 47) | Hydrolase activity, hydrolyzing O-glycosyl compounds | Carbohydrate metabolic process | - |
| **Bra038754** | Tom1, Attom1; Tom1 (Tobamovirus multiplication 1) | Protein binding | - | - |
| **Bra038753** | Msrb2; Msrb2 (Methionine sulfoxide reductase B 2) | Peptide-methionine-(S)-S-oxide reductase activity | Oxidation reduction | - |
| **Bra038749** | Mate efflux family protein | Antiporter activity; drug transporter activity | Transmembrane transport; multidrug transport | Membrane; cellular component |
| **Bra038748** | Mate efflux family protein | Antiporter activity; drug transporter activity | Transmembrane transport; multidrug transport | Membrane; cellular component |
| **Bra019413** | Disease resistance protein (TIR-NBS-LRR class), putative | ATP binding; protein binding | Apoptosis | - |
| **Bra019412** | Disease resistance protein (TIR-NBS-LRR class), putative | Transmembrane receptor activity | Innate immune response; signal transduction | Intrinsic to membrane; cellular component |
| **Bra019410** | Disease resistance protein (TIR-NBS-LRR class), putative | Nucleoside-triphosphatase activity; ATP binding; protein binding; transmembrane receptor activity; nucleotide binding | Innate immune response; signal transduction; apoptosis | Intrinsic to membrane; cellular component |
| **Bra019409** | Disease resistance protein (TIR-NBS-LRR class), putative | ATP binding; protein binding; transmembrane receptor activity | Innate immune response; signal transduction; apoptosis | Intrinsic to membrane; cellular component |
| **Bra019407** | Apg8a, Atg8a; Apg8a (Autophagy 8a); Apg8 activating enzyme/ Apg8-specific protease/ Atg8 ligase | - | - | - |
| **Bra019406** | Apr3, Prh-26, Prh26, Atapr3; Apr3 (Aps reductase 3); adenylyl-sulfate reductase | Transferase activity | Cell redox homeostasis; metabolic process | - |
| **Bra019405** | F-box family protein | - | - | - |
| **Bra019403** | Atmrp1, Est1, Atabcc1, Mrp1; Mrp1 (*A. thaliana* multidrug resis-tance-associated protein 1) | - | - | - |
| **Bra019400** | F-box family protein | - | - | - |
| **Bra019398** | aspartic-type endopeptidase | Aspartic-type endopeptidase activity | proteolysis | - |
| **Bra019395** | DNA binding / protein binding / zinc ion binding | Zinc ion binding; protein binding; DNA binding |  |  |
| **Bra019391** | zinc finger (C3HC4-type RING finger) family protein | Zinc ion binding; protein binding | - | - |
| **Bra019390** | unknown protein | - | - | - |

^a^Information was obtained from <http://brassicadb.org/brad/index.php>. “-” indicates that no information is available
