# Supplementary material for: Identification of Genome-Wide Variants and Discovery of Variants Associated with Brassica rapa Clubroot Resistance Gene Rcr1 through Bulked Segregant RNA Sequencing
Source: PLoS One. 2016 Apr 14;11(4):e0153218. doi: 10.1371/journal.pone.0153218 (PMC4831815; doi:10.1371/journal.pone.0153218)
Supplement: S3 Table — (DOCX) [file pone.0153218.s004.docx]

**S3 Table. Polymorphic variants identified from R bulks in the TIR-NBS-LRR genes.**

| Position | Ref base | Variant base ^a^ | Type | Impact^b^ | Variants in R (%) | Gene name | Depth | | KASP SNP name |
| --- | --- | --- | --- | --- | --- | --- | --- | --- | --- |
|  |  |  |  |  |  |  | R | S |  |
| 24352220 | C | - | InDel | F | 79.0 | Bra019413 | 81 | 53 |  |
| 24352616 | C | A | SNP | S | 30.8 | Bra019413 | 227 | 6 | SNP_A03_11 |
| 24352908 | C | G | SNP | NS | 46.8 | Bra019413 | 237 | 10 | SNP_A03_104 |
| 24353167 | C | T | SNP | NS | 64.2 | Bra019413 | 81 | 12 |  |
| 24353172 | T | A | SNP | NS | 62.0 | Bra019413 | 50 | 13 |  |
| 24353183 | C | T | SNP | S | 21.1 | Bra019413 | 57 | 32 |  |
| 24353191 | C | G | SNP | NS | 24.0 | Bra019413 | 50 | 38 |  |
| 24353220 | C | T | SNP | NS | 25.0 | Bra019413 | 56 | 43 |  |
| 24353223 | C | A | SNP | NS | 26.3 | Bra019413 | 57 | 43 |  |
| 24353289 | T | A | SNP | NS | 28.3 | Bra019413 | 113 | 37 | SNP_A03_08 |
| 24353306 | C | A | SNP | S | 32.7 | Bra019413 | 55 | 22 |  |
| 24353312 | - | T | InDel | F | 23.1 | Bra019413 | 26 | 14 |  |
| 24353761 | G | C | SNP | S | 25.0 | Bra019413 | 20 | 5 |  |
| 24370694 | T | C | SNP | NS | 22.9 | Bra019412 | 35 | 21 |  |
| 24371044 | A | T | SNP | NS | 75.4 | Bra019412 | 407 | 131 | SNP_A03_09 |
| 24374356 | T | A | SNP | NS | 72.5 | Bra019410 | 120 | 34 |  |
| 24374412 | C | T | SNP | NS | 66.4 | Bra019410 | 116 | 37 |  |
| 24374460 | C | G | SNP | NS | 45.5 | Bra019410 | 101 | 19 |  |
| 24374482 | G | C | SNP | NS | 36.4 | Bra019410 | 55 | 10 |  |
| 24374483 | C | T | SNP | S | 75.0 | Bra019410 | 52 | 10 |  |
| 24374496 | G | T | SNP | NS | 71.4 | Bra019410 | 21 | 7 |  |
| 24374730 | A | G | SNP | NS | 57.1 | Bra019410 | 42 | 24 |  |
| 24374852 | A | T | SNP | NS | 26.9 | Bra019410 | 26 | 11 |  |
| 24374878 | G | T | SNP | NS | 100.0 | Bra019410 | 40 | 11 |  |
| 24374928 | G | A | SNP | NS | 100.0 | Bra019410 | 48 | 8 |  |
| 24374961 | C | A | SNP | NS | 68.4 | Bra019410 | 95 | 26 |  |
| 24375018 | T | T | SNP | NS | 40.6 | Bra019410 | 101 | 18 | SNP_A03_102 |
| 24375020 | G | A | SNP | S | 40.2 | Bra019410 | 102 | 16 |  |
| 24375087 | A | C | SNP | NS | 23.1 | Bra019410 | 91 | 19 |  |
| 24375147 | A | G | SNP | NS | 48.3 | Bra019410 | 29 | 11 |  |
| 24375156 | G | C | SNP | NS | 95.7 | Bra019410 | 23 | 12 |  |
| 24375161 | G | C | SNP | NS | 87.5 | Bra019410 | 16 | 10 |  |
| 24375394 | T | A | SNP | NS | 55.2 | Bra019410 | 96 | 57 |  |
| 24375572 | G | C | SNP | NS | 43.1 | Bra019410 | 72 | 32 | SNP_A03_12 |
| 24375579 | A | T | SNP | NS | 43.7 | Bra019410 | 71 | 28 |  |
| 24375757 | A | G | SNP | S | 41.9 | Bra019410 | 31 | 14 |  |
| 24375828 | T | C | SNP | NS | 47.6 | Bra019410 | 63 | 36 |  |
| 24375901 | C | A | SNP | NS | 50.0 | Bra019410 | 60 | 41 |  |
| 24375920 | G | T | SNP | N | 36.7 | Bra019410 | 79 | 42 |  |
| 24375950 | T | C | SNP | NS | 43.1 | Bra019410 | 102 | 42 |  |
| 24375979 | T | C | SNP | S | 46.6 | Bra019410 | 118 | 40 | SNP_A03_13 |
| 24375985 | C | A | SNP | S | 45.5 | Bra019410 | 112 | 38 |  |
| 24376033 | G | A | SNP | S | 53.4 | Bra019410 | 73 | 36 |  |
| 24376036 | G | A | SNP | S | 46.9 | Bra019410 | 64 | 36 |  |
| 24376087 | T | C | SNP | S | 21.1 | Bra019410 | 38 | 40 |  |
| 24376507 | C | C | SNP | S | 46.5 | Bra019410 | 71 | 34 | SNP_A03_14 |
| 24376572 | G | C | SNP | NS | 28.0 | Bra019410 | 50 | 30 |  |
| 24376628 | A | T | SNP | NS | 81.8 | Bra019410 | 110 | 30 |  |
| 24378506 | C | G | SNP | NS | 43.4 | Bra019410 | 53 | 23 |  |
| 24378509 | G | A | SNP | S | 40.4 | Bra019410 | 57 | 24 |  |
| 24378535 | C | T | SNP | NS | 25.4 | Bra019410 | 71 | 43 |  |
| 24378671 | G | A | SNP | NS | 36.7 | Bra019410 | 60 | 39 | SNP_A03_103 |
| 24378759 | C | T | SNP | S | 13.7 | Bra019410 | 300 | 145 |  |
| 24378856 | T | C | SNP | NS | 40.1 | Bra019410 | 307 | 94 |  |
| 24378971 | G | A | SNP | NS | 51.2 | Bra019410 | 86 | 37 |  |
| 24379049 | T | G | SNP | NS | 44.7 | Bra019410 | 85 | 35 |  |
| 24379080 | C | A | SNP | NS | 45.6 | Bra019410 | 68 | 32 |  |
| 24381766 | A | C | SNP | NS | 52.7 | Bra019409 | 55 | 39 |  |
| 24381881 | T | A | SNP | NS | 63.7 | Bra019409 | 102 | 61 |  |
| 24381943 | G | C | SNP | S | 70.7 | Bra019409 | 133 | 65 |  |
| 24381952 | A | G | SNP | S | 48.9 | Bra019409 | 133 | 72 |  |
| 24381985 | T | C | SNP | S | 55.5 | Bra019409 | 110 | 63 |  |
| 24382012 | T | C | SNP | S | 65.2 | Bra019409 | 89 | 64 | SNP_A03_16 |
| 24382158 | C | T | SNP | NS | 100.0 | Bra019409 | 20 | 28 |  |
| 24382295 | G | A | SNP | NS | 100.0 | Bra019409 | 9 | 31 |  |
| 24382298 | G | T | SNP | NS | 100.0 | Bra019409 | 9 | 32 |  |
| 24382300 | T | C | SNP | S | 100.0 | Bra019409 | 9 | 35 |  |
| 24382382 | G | T | SNP | NS | 100.0 | Bra019409 | 21 | 38 |  |
| 24382408 | C | A | SNP | NS | 100.0 | Bra019409 | 7 | 37 |  |
| 24382482 | G | T | SNP | NS | 100.0 | Bra019409 | 6 | 34 |  |
| 24382587 | C | T | SNP | NS | 100.0 | Bra019409 | 36 | 19 | SNP_A03_101 |
| 24382594 | G | A | SNP | NS | 100.0 | Bra019409 | 35 | 17 |  |
| 24382636 | C | A | SNP | N | 100.0 | Bra019409 | 27 | 21 |  |
| 24382651 | C | A | SNP | S | 100.0 | Bra019409 | 16 | 24 |  |
| 24382691 | G | A | SNP | NS | 90.9 | Bra019409 | 22 | 27 |  |
| 24382703 | G | A | SNP | NS | 100.0 | Bra019409 | 19 | 28 |  |
| 24383077 | T | - | InDel | F | 26.6 | Bra019409 | 139 | 61 |  |
| 24383198 | G | A | SNP | NS | 100.0 | Bra019409 | 24 | 38 |  |
| 24383256 | G | A | SNP | NS | 100.0 | Bra019409 | 14 | 51 |  |
| 24383290 | A | G | SNP | S | 100.0 | Bra019409 | 10 | 59 |  |
| 24383456 | G | A | SNP | NS | 87.5 | Bra019409 | 8 | 36 |  |
| 24383732 | T | A | SNP | N | 100.0 | Bra019409 | 11 | 48 |  |
| 24383733 | T | C | SNP | NS | 100.0 | Bra019409 | 11 | 48 |  |
| 24383817 | C | T | SNP | N | 26.9 | Bra019409 | 67 | 70 |  |
| 24383964 | T | C | SNP | NS | 100.0 | Bra019409 | 8 | 41 |  |
| 24383986 | A | G | SNP | S | 100.0 | Bra019409 | 11 | 36 |  |
| 24384110 | A | G | SNP | NS | 33.3 | Bra019409 | 27 | 45 |  |
| 24384138 | A | G | SNP | NS | 32.1 | Bra019409 | 56 | 88 |  |
| 24384191 | A | T | SNP | NS | 33.3 | Bra019409 | 9 | 38 |  |
| 24384928 | T | C | SNP | S | 100.0 | Bra019409 | 5 | 43 |  |
| 24384947 | A | G | SNP | NS | 100.0 | Bra019409 | 18 | 41 |  |
| 24385013 | G | A | SNP | NS | 23.5 | Bra019409 | 68 | 54 |  |
| 24385048 | T | C | SNP | S | 25.8 | Bra019409 | 31 | 52 |  |
| 24385049 | T | C | SNP | NS | 100.0 | Bra019409 | 26 | 54 |  |
| 24385061 | A | C | SNP | NS | 25.0 | Bra019409 | 12 | 58 |  |
| 24385066 | A | T | SNP | S | 40.0 | Bra019409 | 10 | 60 |  |
| 24385116 | G | A | SNP | NS | 100.0 | Bra019409 | 9 | 37 |  |
| 24385126 | C | T | SNP | S | 100.0 | Bra019409 | 9 | 31 |  |
| 24385788 | A | G | SNP | NS | 62.5 | Bra019409 | 8 | 89 |  |
| 24385806 | A | G | SNP | NS | 62.5 | Bra019409 | 8 | 67 |  |
| 24385807 | C | T | SNP | NS | 100.0 | Bra019409 | 8 | 87 |  |
| 24385918 | A | G | SNP | NS | 100.0 | Bra019409 | 47 | 101 |  |
| 24385942 | C | T | SNP | NS | 97.3 | Bra019409 | 73 | 100 |  |
| 24385953 | G | A | SNP | NS | 98.6 | Bra019409 | 72 | 99 |  |
| 24385976 | T | C | SNP | S | 100.0 | Bra019409 | 55 | 97 |  |
| 24385996 | T | A | SNP | NS | 100.0 | Bra019409 | 37 | 98 |  |
| 24386298 | - | A | InDel | F | 100.0 | Bra019409 | 8 | 59 |  |
| 24386300 | - | G | InDel | F | 100.0 | Bra019409 | 8 | 59 |  |
|  |  |  |  |  |  |  |  |  |  |

^a^ “-”indicates insertion or deletion.

^b^ Variants that affect amino acid sequences into four groups: non-synonymous (NS), nonsense (N), frameshift (F) and synonymous (S)
