# Supplementary material for: Identification of Genome-Wide Variants and Discovery of Variants Associated with Brassica rapa Clubroot Resistance Gene Rcr1 through Bulked Segregant RNA Sequencing
Source: PLoS One. 2016 Apr 14;11(4):e0153218. doi: 10.1371/journal.pone.0153218 (PMC4831815; doi:10.1371/journal.pone.0153218)
Supplement: S4 Table — (DOCX) [file pone.0153218.s005.docx]

### **S4 Table. SNPs and their flanking sequences^a^**

^a^SNP s are shown in brackets.
